# Confocal Laser Endomicroscopy Assessment of Pituitary Tumor Microstructure: A Feasibility Study

**DOI:** 10.3390/jcm9103146

**Published:** 2020-09-29

**Authors:** Evgenii Belykh, Brandon Ngo, Dara S. Farhadi, Xiaochun Zhao, Michael A. Mooney, William L. White, Jessica K. Daniels, Andrew S. Little, Jennifer M. Eschbacher, Mark C. Preul

**Affiliations:** 1 The Loyal and Edith Davis Neurosurgical Research Laboratory, Department of Neurosurgery, Barrow Neurological Institute, St. Joseph’s Hospital and Medical Center, Phoenix, AZ 85013, USA; belykhevgenii@gmail.com (E.B.); bqn@email.arizona.edu (B.N.); darafarhadi@email.arizona.edu (D.S.F.); kyle.g0704@gmail.com (X.Z.); mmooney6@bwh.harvard.edu (M.A.M.); william.white@barrowbrainandspine.com (W.L.W.); andrew.little@barrowbrainandspine.com (A.S.L.); 2 Department of Neuropathology, Barrow Neurological Institute, St. Joseph’s Hospital and Medical Center, Phoenix, AZ 85013, USA; jessica.daniels@dignityhealth.org (J.K.D.); jennifer.eschbacher@dignityhealth.org (J.M.E.)

**Keywords:** confocal endomicroscopy, fluorescein sodium, frozen section, histology, pituitary, tumor

## Abstract

This is the first study to assess confocal laser endomicroscopy (CLE) use within the transsphenoidal approach and show the feasibility of obtaining digital diagnostic biopsies of pituitary tumor tissue after intravenous fluorescein injection. We confirmed that the CLE probe reaches the tuberculum sellae through the transnasal transsphenoidal corridor in cadaveric heads. Next, we confirmed that CLE provides images with identifiable histological features of pituitary adenoma. Biopsies from nine patients who underwent pituitary adenoma surgery were imaged ex vivo at various times after fluorescein injection and were assessed by a blinded board-certified neuropathologist. With frozen sections used as the standard, pituitary adenoma was diagnosed as “definitively” for 13 and as “favoring” in 3 of 16 specimens. CLE digital biopsies were diagnostic for pituitary adenoma in 10 of 16 specimens. The reasons for nondiagnostic CLE images were biopsy acquisition <1 min or >10 min after fluorescein injection (*n* = 5) and blood artifacts (*n* = 1). In conclusion, fluorescein provided sufficient contrast for CLE at a dose of 2 mg/kg, optimally 1–10 min after injection. These results provide a basis for further in vivo studies using CLE in transsphenoidal surgery.

## 1. Introduction

The standard method for rapid intraoperative diagnosis in pituitary surgery is frozen section histopathological analysis. This technique relies on the evaluation of limited amounts of tissue to help guide surgical decision making. Despite its role in intraoperative diagnosis, frozen section analysis has marked limitations, including the need for processing, preparation time, artifacts, and decreased availability of tissues for permanent specimen analysis [1,2]. Although permanent sections offer higher diagnostic accuracy than frozen sections, permanent section analysis requires even longer processing times [3]. Real-time guidance of pituitary adenoma resection requires more rapid tissue analysis than classic histologic interpretation provides.

Fluorescence imaging, such as confocal laser endomicroscopy (CLE), which yields real-time imaging, is a faster modality of data acquisition than frozen or permanent section analysis, and it can be used intraoperatively to improve surgical decision making. CLE is essentially a miniaturized (i.e., the size of a neurosurgical suction tip), portable, handheld, fiber optic-based, confocal laser scanning microscope used in conjunction with a 488-nm excitable fluorophore. This fluorophore emits energy that is detectable by the system. The instrument’s lens at the tip of the probe is scanned across or held steady on the surface of the target tissue while the system generates on-the-fly fluorescence images of the tissue with a level of resolution that shows cellular detail. CLE is advantageous because it allows for rapid (real-time) image analysis by the surgeon and pathologist from different observational perspectives, including remote vantage points [4,5].

The effectiveness of this technique was first demonstrated in gastrointestinal and gynecological operations; in those studies, it was shown to increase the diagnostic yield of biopsies and decrease the need for numerous biopsies [6,7]. CLE practicability has been examined extensively in a murine malignant glioma model, and its real-time diagnostic application in human brain tumor resection has been assessed and established [8,9,10,11]. Furthermore, the diagnostic specificities and sensitives of CLE for gliomas and meningiomas were similar to those of frozen sections [12]. The main uses of CLE in neurosurgery are to optimize the resection margin of invasive lesions and to detect abnormal or tumor tissue in suspect resection areas. Clinical-grade CLE systems have been approved for use in gastroenterology [13], pulmonology [14], and other specialties, and one system has recently been approved by the FDA for use in the brain. We have assessed this system at our institution for its performance for use in brain tumors ex vivo but still using the system for immediate tissue assessment within the operating room, and we are now conducting in vivo feasibility testing during surgery. Such testing has largely been aimed at brain tumors comprising those that do not commonly originate in the sella turcica or in association with the pituitary gland, such as gliomas, meningiomas, and metastases.

However, the CLE system was also used in pituitary cases, and thus this study represents an application in a subset of pathology that may be unusual and indicate potential for the use of CLE in diagnosis and surgical assessment. The feasibility of pituitary tissue and lesion or mass assessment with CLE has not been examined. This study examines, to the best of our knowledge for the first time, the feasibility of CLE, with fluorescein used as a fluorescence contrast agent, for rapid intraoperative assessment of the tissue microstructure of human pituitary adenomas.

## 2. Materials and Methods

### 2.1. Feasibility of CLE Positioning for Endonasal Procedures

Three cadaveric heads with arterial and venous systems injected with colored silicone were used to assess the endonasal placement of the scanning probe in the pituitary gland. An endonasal transsphenoidal approach to the pituitary gland was established with the use of the standard endoscopic instruments and a straight endoscope.

### 2.2. Biopsy Specimens

This prospective study was approved by the St. Joseph’s Hospital and Medical Center Institutional Review Board for Human Research (PHXA-10BN130). A total of 9 patients who underwent endonasal transsphenoidal surgery for pituitary adenoma within a 3-month period were included. Fluorescein was administered intravenously (IV) at a dose of 2 mg/kg before biopsy collection. Tissue specimens that were suspected to contain a pituitary tumor and were to be removed as a usual part of the standard procedure were collected for this study. These biopsy specimens were not used for patient care decision making and were acquired when sufficient tissue was available after permanent and frozen section biopsy specimens were acquired for patient care. Immediately after resection, specimens were put on a moisturized nonadherent surgical dressing and examined ex vivo in the same operating room but away from the patient. A confocal laser endomicroscope (CONVIVO, Carl Zeiss AG, Jena, Germany) was used to obtain confocal digital images. Time to imaging the tissue specimen following fluorescein injection ranged from 1 to 45 min.

### 2.3. Imaging Protocol

CLE uses data acquisition parameters comparable to those presented in our previous work [8,15]. The CLE system includes a 15-cm-long scanner probe shaft covered by a sterile outer sheath (5-mm outer diameter). This probe is rigidly connected to a scanner unit using a 3.8-m-long cable. The scanner unit itself is attached to a mobile workstation. The scanning depth and recording functions are controlled through a touchscreen display or controlled remotely to vary imaging from the surface to a depth of 0–500 μm. Incident excitation light is generated by a 488-nm laser. A single optical fiber serves as both the excitation and detection pinholes for confocal isolation of the focal plane. The detector signal and scanning are digitally synchronized to produce en face optical sections creating images parallel to the tissue surface. Aside from the IV fluorescein, no additional fluorophore was used for ex vivo imaging.

To create optimal-quality images, we set the system to use a green bandpass filter (517.5–572.5 nm) to detect emitted fluorescence. Generally, the laser power setting was set at 0.5–0.9 mW during the scanning, with a maximum power limit of 1 mW. Using 1× zoom, we scanned a field of view of 267 × 475 μm at 1920 × 1080 pixels (full high definition) with a speed of 0.75 frames per second and with lateral and axial resolutions of approximately 0.7 and 4.5 μm, respectively. The static images produced were saved and used to create a time-lapse series that functioned as a digital looping video.

Trained team members performed the CLE scanning (EB, XZ). Digital optical biopsies were primarily performed with the CLE probe affixed in the upright position to a retractor arm that was tightened to stabilize the probe position. Small tissue specimens (area, 2–5 mm^3^) were placed on the surface of the probe ex vivo immediately after acquisition and, while maintaining contact with the probe, were gently moved with microsurgical forceps to scan the larger sample surface. Multiple images were acquired for every biopsy location.

### 2.4. Tissue Sampling, Histology, and Processing

After CLE imaging, each biopsy specimen was divided in half for frozen section and permanent section preparation and sent to the pathology department. Histologic analysis was conducted using standard light microscopic evaluation of 10-μm-thick hematoxylin and eosin (H&E)-stained sections. Both frozen and permanent histologic sections were used to validate the CLE images. The reason for using H&E staining as a reference standard rather than other specific stains was that, similarly to fluorescein, H&E provides nonspecific staining of both normal and abnormal tissue. Furthermore, CLE is an in vivo tool for preliminary histological evaluation of the tissue that complements or gives an alternative to frozen section analysis.

A neuropathologist with experience interpreting CLE images, but who was not involved in the surgical procedures, reviewed the CLE digital images (several images and video loops from one specimen), as well as frozen and permanent section slides. An educational set of CLE images was reviewed before examining the study CLE images. Histology slides and CLE images were presented in random order. Histopathologic features of the CLE images and H&E-stained frozen and permanent sections were assessed and rated using a questionnaire (Supplement 1). CLE images were categorized as diagnostic if they showed identifiable histologic features.

## 3. Results

### 3.1. Feasibility of CLE Positioning for Transsphenoidal Surgery

**Figure 1:**
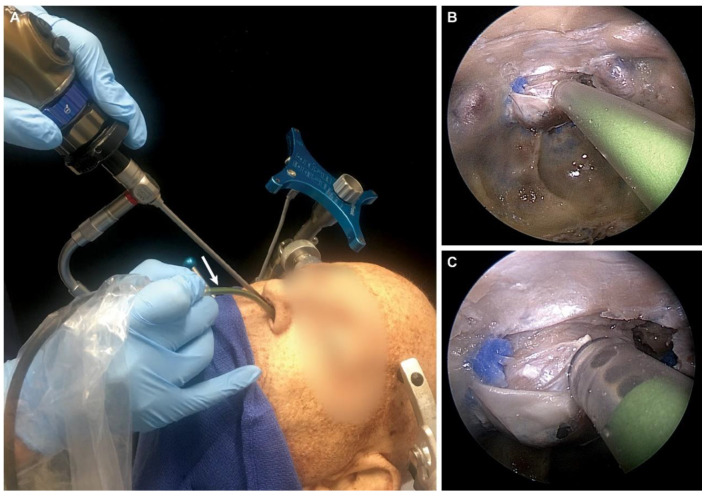
Position of confocal laser endomicroscopy (CLE) scanning probe in the endonasal transsphenoidal approach. (**A**) Overall position (arrow points to the CLE probe). (**B**) Far and (**C**) close-up endoscopic view of the tip of the probe in the sellar region. Used with permission from Barrow Neurological Institute, Phoenix, Arizona.

The scanning probe was successfully used in a transsphenoidal approach in all three cadaveric specimens. The size of the probe was adequate for en face imaging of the pituitary gland tissue within the sella turcica (Figure 1).

### 3.2. Descriptive Analysis

**Figure 2:**
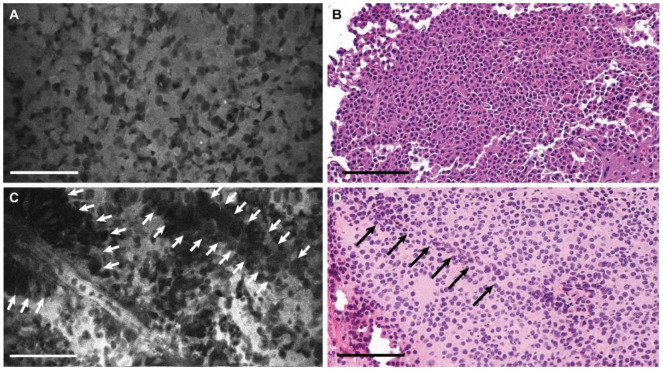
Photomicrographs of a pituitary adenoma. (**A**) Confocal laser endomicroscopy (CLE) image (see Supplementary Video S1) and (**B**) hematoxylin and eosin (H&E) stain of the same tumor showing sheets of uniform nonlobulated cells with prominent nuclei. (**C**) CLE image (see Supplementary Video S2) and (**D**) H&E image showing perivascular sheets of the cells (*arrows*). Bar = 100 μm. Used with permission from Barrow Neurological Institute, Phoenix, Arizona.

The CLE images of pituitary adenomas demonstrated appropriate characteristic features: sheets of cells with prominent nuclei, increased cellularity, nonorganized tissue architecture, vascularity, and stroma (Figure 2 and Supplementary Videos S1 and S2). We observed heterogenous uptake of fluorescein by cells creating a nuclear–cytoplasmic contrast, as well as contrast between neighboring cells.

### 3.3. Blinded Review

**Figure 3:**
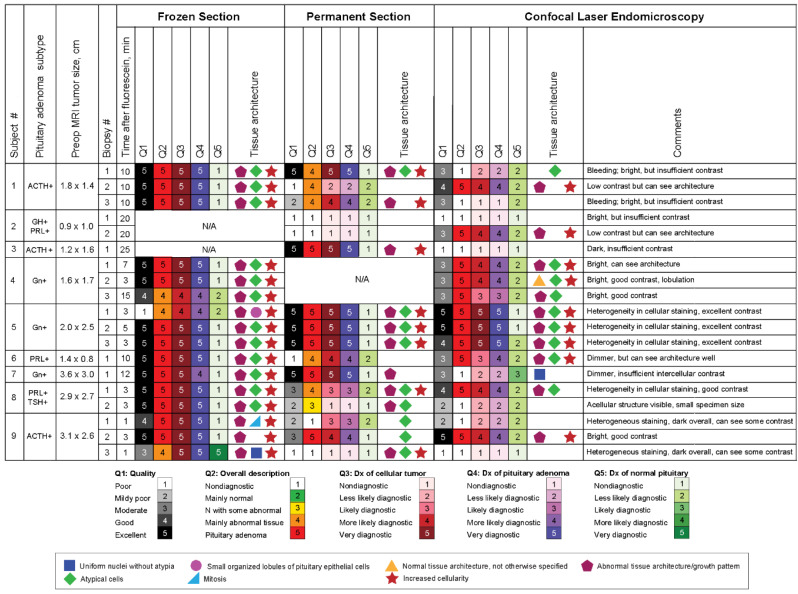
Results of a qualitative comparative assessment of frozen sections, fixed permanent sections, and confocal laser endomicroscopy images of pituitary adenomas by a neuropathologist blinded to patient data. Abbreviations: ACTH, adrenocorticotropic hormone; Dx, diagnosis; GH, growth hormone; Gn, gonadotroph; N, normal; N/A, not applicable; PRL, prolactin; TSH, thyroid stimulating hormone. Used with permission from Barrow Neurological Institute, Phoenix, Arizona.

**Figure 4:**
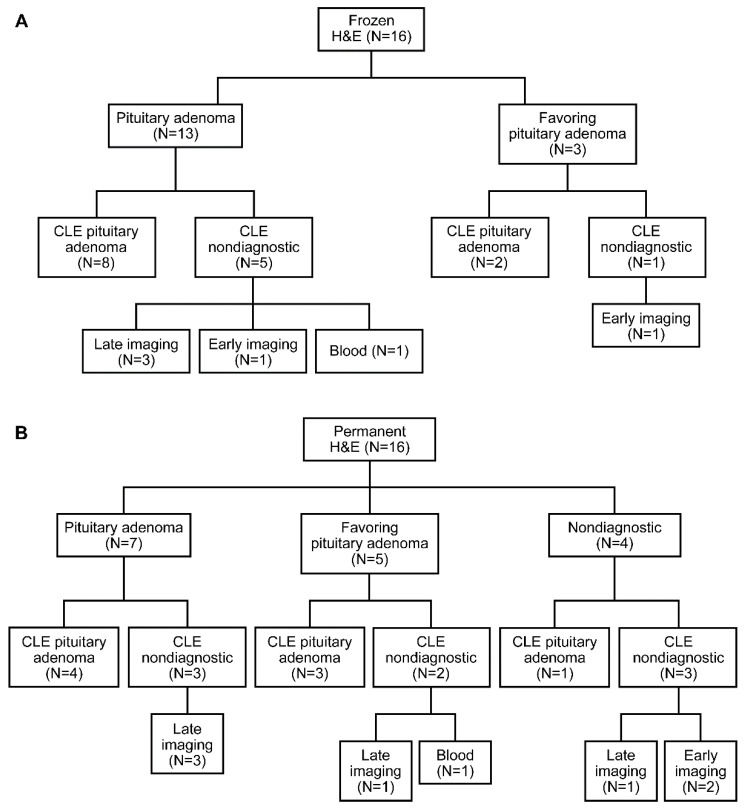
Study design flowchart showing confocal laser endomicroscopy (CLE) accuracy with (**A**) frozen hematoxylin and eosin (H&E) sections and (**B**) fixed permanent H&E sections used as the standard. Used with permission from Barrow Neurological Institute, Phoenix, Arizona.

**Figure 5:**
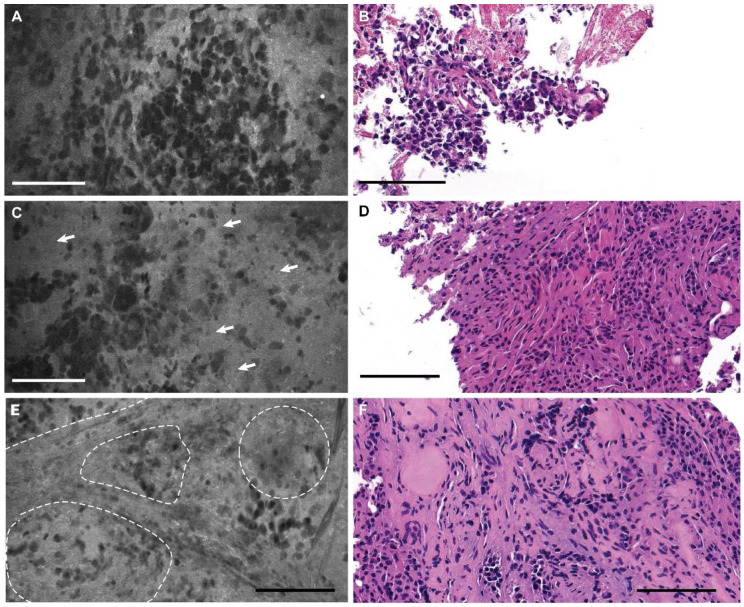
(**A**) Confocal laser endomicroscopy (CLE) image that was nondiagnostic because the biopsy specimen was acquired too early (<1 min after fluorescein injection); (**B**) a hematoxylin and eosin (H&E)-stained section from the same specimen. (**C**) CLE image that was nondiagnostic because the biopsy specimen was acquired too late (>10 min after fluorescein injection), with arrows pointing to the hypercellular areas of tissue with suboptimal contrasting of the cellular outlines; (**D**) an H&E-stained section from the same specimen. (**E**) CLE image showing more uniform lobules of pituitary epithelial cells, suggestive of normal pituitary tissue, with dotted lines outlining lobules; (**F**) H&E-stained section from the same specimen. Bars = 100 μm. Used with permission from Barrow Neurological Institute, Phoenix, Arizona.

The study included nine patients with pituitary adenomas that resulted in 19 specimens scanned with CLE. Results of the blinded review are presented in Figure 3. A neuropathologist and pathologist correctly identified 8 of 13 pituitary adenomas imaged with CLE using frozen sections as the comparative diagnostic standard, whereas the remaining five specimens scanned with CLE that correlated with frozen section-identified adenomas were considered nondiagnostic (Figure 4). Furthermore, among the seven sections identified as being obtained from pituitary adenomas using permanent sections as the standard, four were also identified as being from pituitary adenomas using CLE, whereas the other three specimens were deemed nondiagnostic. Nondiagnostic CLE images were attributed to the timing of fluorescein injection and erythrocytes in the field that obscured useful interpretation. Most nondiagnostic CLE images were associated with biopsies that were acquired very early (<1 min) or very late (>10 min) after fluorescein was injected (Figure 5). Additionally, very small biopsy specimens made it difficult to find optimal imaging locations. Blood present in one specimen was characterized by the neuropathologist as nondiagnostic. When examining histological and CLE information from patients, frozen section, permanent section, and CLE analysis were able to successfully identify a tumor in 7/7, 7/8, and 9/9 cases, respectively.

## 4. Discussion

### 4.1. Confocal Endomicroscopy Technology

Although frozen sections and imprint cytology [16] are routine techniques used for intraoperative diagnosis, they are not infallible. These histologic analyses are subject to preparation and processing time delays, freezing and sectioning artifacts, and potential tissue loss and degradation. Furthermore, diagnostic discrepancies exist between frozen and permanent sections [3]. Studies have suggested that confocal reflectance microscopy is an effective alternative to use in standard histologic analysis for intraoperative diagnosis and evaluation of tumor margins [17,18,19,20]. Another reflectance microscopy technology based on stimulated Raman scattering was shown to be effective for digital biopsies of ex vivo specimens of lesional brain tissue [21].

Unlike the above-mentioned technologies, CLE is intended for handheld, portable, in vivo digital optical interrogation of tissues without the need for tissue removal. A previous generation of the CLE device was determined to be an effective diagnostic imaging modality in a study of multiple intracranial tumors, including gliomas and meningiomas [12]. CLE technology has also been successfully used to visualize internal tissue microstructures, such as fiber bundles and cellular and nuclear shapes and sizes, in multiple brain tumor types [22,23,24].

In this study, we performed a proof-of-principle assessment of pituitary adenoma tissue with the first clinical-grade and US Food and Drug Administration-approved CLE device. CLE imaging allows for the spatial resolution of biopsy specimens without physical disturbance. When combined with the appropriate fluorescent dye, CLE can be effective in visualizing characteristic tissue architecture and morphology for histopathologic analysis. Moreover, the CLE probe is similar in size to a standard neurosurgical suction device, permitting manual application in real-time intraoperative visualization of brain tumors and normal tissue at the cellular level [25].

This proof-of-principle study has demonstrated the potential use of CLE in detecting the pituitary adenoma tissue microstructure. A neuropathologist with no prior knowledge of the biopsied specimens was able to correctly identify 8 of 13 CLE images (62%) as indicating pituitary adenomas using frozen sections as the standard. The remaining five images were labeled as nondiagnostic for the following reasons: three were imaged very late (>10 min after fluorescein injection), one was imaged very early (<1 min after fluorescein injection), and one showed too much blood artifact. Future studies should use tissue acquisition between 1 and 10 min after fluorescein injection to reduce nondiagnostic image collection. In vivo imaging should allow for better CLE quality due to a larger area being available for scanning, which would permit diagnostic frame detection. Nevertheless, bleeding artifacts would still remain.

### 4.2. Biopsy Acquisition

The small size of some pituitary biopsy specimens presented an obstacle for an adequate histological assessment, which should be noted for future study planning. At a patient level, all permanent diagnoses agreed with the frozen section diagnosis. At the biopsy level, all but one of the frozen sections were associated with a diagnosis of “very likely” pituitary adenoma, and one frozen section was described as “mainly abnormal tissue.” Two permanent sections had inconclusive findings, and one permanent section contained both normal and abnormal tissue. Although we believe that some CLE biopsy specimens exhibited the lobulated appearance characteristic of normal pituitary architecture (Figure 5E,F), none of the histological slide specimens were composed completely of normal pituitary gland tissue. Therefore, future in vivo CLE assessments will need to confirm the histological appearance of normal pituitary gland tissue.

### 4.3. Optimal Dosing and Timing of Fluorescein Injection

The optimal timing for CLE imaging was determined to be more than 1 min but less than 10 min after fluorescein injection. For this time frame, 2 mg/kg IV fluorescein was a sufficient dosing regimen, but other doses have yet to be evaluated for pituitary adenomas. In another study, a 5-mL IV dose of 10% fluorescein was used to visualize gliomas and meningiomas [12]. The dosing of fluorescein is still somewhat undefined or based on empirical evidence. In our experience, fluorescein may be readministered a few minutes before the surgical lesion or region is assessed with CLE that may, in turn, lead to further manipulation or resection of tissue. The successful use of a higher dose to visualize other brain neoplasms warrants further investigation as to whether a similar approach would improve CLE imaging of pituitary masses.

### 4.4. Feasibility for Endonasal Procedures

**Figure 6:**
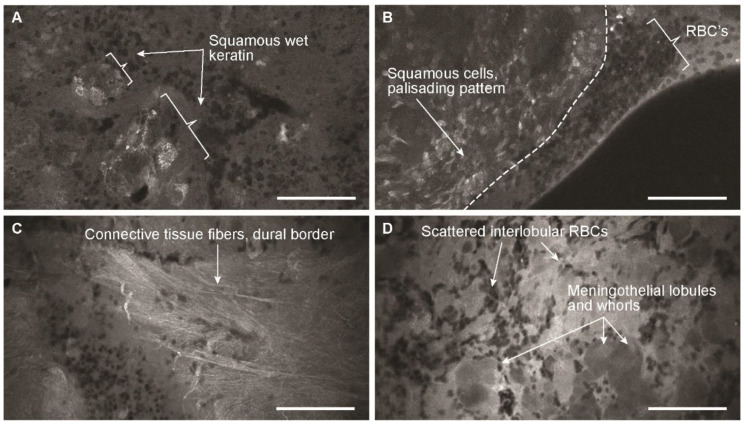
Confocal laser endomicroscopy (CLE) images of (**A**,**B**) craniopharyngioma and (**C**,**D**) meningioma. Bars = 100 μm. RBCs, red blood cells. Used with permission from Barrow Neurological Institute, Phoenix, Arizona.

CLE offers a potential imaging modality for minimally invasive transnasal assessment of skull base and pituitary lesions. However, the CLE probe design was previously too wide in its shaft diameter, which did not allow for transnasal access. The new, more refined, and miniaturized CLE probe design that has been approved by the FDA easily allows access through the transnasal transsphenoidal corridor. We believe that CLE may be useful for managing certain patients with pituitary lesions and sellar masses of unclear origin. CLE could be used to positively identify the tumor margin in questionable regions. It could also be used for preliminary diagnosis, similar to a frozen section, or to increase the positive yield of a viable and informative tissue sample acquisition for histological analysis. CLE could be used to differentiate lesions when the previous diagnostic work-up, including MRI and laboratory results for pituitary hormone levels, serum angiotensin-converting enzyme levels (to identify sarcoidosis), or hCG levels (to identify a germinoma), does not provide sufficient evidence for a clear diagnosis. In addition, CLE could serve as an adjunct for intraoperative differential diagnosis of sellar mass lesions of uncertain origin, such as meningiomas, craniopharyngiomas, inflammatory lesions, and metastases. Although the current study was designed to evaluate tissue samples taken from the pituitary tissue, our previous experience with imaging of craniopharyngioma and meningioma tissues suggested that these tumor types could be differentiated on CLE (Figure 6). The potential benefits of CLE compared to traditional intraoperative tissue evaluation include its noninvasive nature, the ability to assess multiple regions, and the increased speed of histoarchitecture image review. This speed provides the surgeon with real-time on-the-fly information and allows immediate communication of images to the pathologist for a preliminary diagnosis or discrimination of abnormal tissue. A proof-of-concept study of intraoperative, transnasal CLE could be conducted, modeled on the study we have conducted here, to evaluate a means of detecting remaining microscopic pathologic tissue remnants.

The concept of a histological “safe margin” of resection is currently not very relevant in pituitary surgery, as the surgeon aims to preserve normal pituitary tissue and to detect cavernous sinus invasion, which usually limits more radical tumor resection. The “safe margin” concept has traditionally been considered relevant for histological assessment in cortical resections, where a detectable margin of tumor to nontumor tissue may be discriminated. Such histological information in pituitary surgery is not present in pathological samples for safety reasons. However, as CLE techniques are refined, they may allow the surgeon to assess whether there has been tumor invasion into margins of the sella turcica or the cavernous sinus. Some of these tumors are now approached anatomically more widely in association with endoscopic neurosurgical procedures.

The goals of pituitary tumor surgery include complete surgical resection with low recurrence rates and preservation of normal pituitary tissue. These goals are particularly relevant for microadenomas in patients affected by Cushing’s disease with a poorly convincing MRI. In such microadenomas, wide-field molecular imaging might be a better adjunct for lesion identification. However, CLE could also be used as a noninvasive tool for histological confirmation of the tissue that is subjected to biopsy or resection. Since the diagnosis of functioning pituitary tumors is usually made preoperatively, the expected role of CLE is to differentiate between tumor and normal pituitary tissue. Such differentiation is difficult on traditional histopathological slides and frequently requires special stains (reticulin and collagen immunostaining) to assess for the fiber network disruption by the tumor, especially when differentiating it from pituitary hyperplasia. Moreover, as was again demonstrated in our study, tissue samples usually include regions of both tumor and normal pituitary tissue. Since CLE provides dynamic in vivo microarchitectural imaging that includes cellular movements and the fibrous extracellular matrix that is lacking on static fixed slides, it is believed that CLE could differentiate such histological variations; however, further in vivo analysis is needed to confirm this supposition.

Although this is a feasibility study, several potential aspects merit discussion. The potential cost of CLE use would include a disposable sterile drape, capital equipment cost for the CLE system, and a procedural fee for interpretation. In fact, such coding has been established for gastrointestinal applications of CLE and is being assessed and defined for neurosurgery. Although we did not assess the time added to the case in this study, our previous prospective assessment of a CLE system demonstrated practical implications for the operating room-to-pathology workflow, including that the first diagnostic frame typically appeared within 17 s after initiation of imaging in vivo [12].

### 4.5. Other Fluorophores

Although this study used fluorescein as the fluorescent dye for CLE, other fluorophores have the potential to aid in the visualization of pituitary adenomas. Other studies have assessed the efficacy of two near-infrared (700–850-nm) fluorophores in pituitary adenoma resections: indocyanine green (ICG) and OTL38 (On Target Laboratories, West Lafayette, IN, USA) [26,27,28]. ICG, a dye frequently used in neurosurgery, can be applied in a technique known as second-window ICG, which uses the enhanced vascular permeability of pituitary adenomas to allow for intraoperative imaging. Additionally, this second-window technique has shown higher sensitivity for all pituitary adenomas than visible-light spectrum visualization alone. OTL38 is a folate ligand combined with an ICG analog that has shown promise in visualizing tumors that overexpress folate receptors, such as nonfunctional pituitary adenomas [29,30]. Both of these dyes have demonstrated increased tissue penetration and a considerably lower background signal when compared with 5-aminolevulinic acid (5-ALA) and fluorescein [31,32]. OTL38 has exhibited higher sensitivity and higher specificity for nonfunctional pituitary adenomas in comparison with white light [33]. Unlike a specific folate dye, fluorescein penetrates the tissue nonspecifically, creating a contrast for better visualization of the contours of all cells and nuclei as well as extracellular fibers. Therefore, unlike other specific dyes, the interpretation is not based solely on the presence of the fluorescent signal but mainly on the evaluation of cytoarchitecture and histological features. Since fluorescein does not interfere with near-infrared ICG and 5-ALA, fluorescein-based CLE is compatible with other wide-field fluorescence-guided surgery techniques used in operating microscopes. Further development of CLE technology with other fluorophores, such as ICG [10,22] and 5-ALA [34], is possible.

### 4.6. Other Imaging Technologies

Other advanced optical imaging technologies that should be mentioned in the context of CLE can be divided into two categories: wide-field fluorescence imaging and small-field confocal imaging. Wide-field fluorescence imaging relies on visualization of unspecific metabolic or targeted fluorescent molecular probes as discussed above; this imaging technique is advantageous for margin detection. Confocal imaging involves a point-scanning excitation laser and a pinhole aperture that filters out-of-focus detected light that allows pixel-by-pixel en face imaging of tissue at a certain depth with submicron resolution [35]. Various technologies that employ the confocal principle include confocal reflectance microscopy, which relies on the detection of reflected excitation light, and confocal fluorescence microscopy, which relies on the detection of a fluorescence signal of specific wavelengths emitted by a fluorophore. We have previously demonstrated the feasibility of confocal reflectance microscopy using a 633-nm laser for histological imaging of pituitary adenoma tissue [17] and other brain tumors [18], but this technology is only available on a relatively larger benchtop confocal microscope system and has not been adopted for portable CLE probe-based in vivo use. When lasers of a higher wavelength are used, multiphoton excitation can be leveraged for the detection of intrinsic tissue fluorophores [36,37] or Raman spectroscopic signatures of molecules [38] to distinguish cellular boundaries and connective tissue and to differentiate normal from tumor tissue. Unlike the above-mentioned technologies, CLE is the only small-field imaging technology available with a compact handheld-sized probe, and there are a few systems already approved for in vivo use in humans [39]. 

### 4.7. Limitations

In our study, the precise diagnosis could not be determined by CLE for every case. Pituitary adenomas that exhibited apparent characteristic features, such as sheets of cells with prominent nuclei, brightly reflective cytoplasm, and increased cellularity, could be identified. However, various factors rendered some specimens nondiagnostic in the neuropathologist’s evaluation. A significant limiting factor that created difficulty in interpreting some of the CLE images, as well as some of the permanent H&E-stained sections, was the small size of the biopsy specimens for ex vivo imaging. This factor should be considered in the future for planning studies.

We have not compared various pituitary adenoma subtypes (subtypes are shown in Figure 1). However, because fluorescein is a nonspecific contrast agent, we do not expect differences. The goal for the CLE assessment is to complement and perhaps replace the conventional intraoperative frozen section analysis performed with nonspecific H&E staining. Other tumor types that present as sellar or parasellar masses, such as germinomas, nontumorous gland hyperplasia, Langerhans histiocytosis, and sarcoidosis, will need to be studied to create a CLE atlas of various tumor types and to further strengthen the role of CLE in the differential diagnosis of parasellar masses.

CLE imaging timing was also a significant factor in the diagnostic capability. Some images acquired more than 10 min or less than 1 min after fluorescein injection were nondiagnostic. Although there were diagnostic images that were acquired 10 min after injection, the proportion of images that were nondiagnostic increased with time. This may be because, as fluorescein was distributed evenly throughout the cells, the contrast between the intracellular, intranuclear, and extracellular compartments diminished. When imaging was obtained too early, the fluorescein was not optimally diffused, and thus cytoplasmic–nuclear contrast was not apparent. Other inconclusive CLE images were obtained from suboptimal locations with the presence of blood artifacts, which impaired the proper image appraisal.

This study did not assess diagnostic accuracy. Here, we mainly assessed the optimal timing and feasibility of the fluorescein dose as well as the feasibility of the CLE probe to be used in the transsphenoidal approach. The probe was not approved for in vivo use at the time that this study was conducted. Investigation of in vivo CLE use is the subject of studies comparing CLE and frozen sectioning for assessing pituitary adenomas, with careful consideration of CLE imaging timing, specimen location, and specimen numbers.

Finally, although fluorescein has a long record of use in ophthalmology and neurosurgery, adverse reactions are possible. However, the estimated incidence of adverse reactions is below 5%, and severe adverse reactions occur in less than 0.01% [40].

## 5. Conclusions

CLE imaging is feasible through a transnasal transsphenoidal corridor for skull base sellar lesions. This pilot study suggests that CLE can be used to visualize histopathologic characteristics of pituitary adenomas, with the added benefit of preserving specimens for permanent section analysis. Fluorescein provided a sufficient contrast for CLE imaging of pituitary adenomas at a dose of 2 mg/kg IV. The optimal imaging interval was 1–10 min after fluorescein injection. Most nondiagnostic CLE images were attributable to limited tissue availability, suboptimal timing, and blood artifacts; these limitations should be taken into account for in vivo use. Additional studies will evaluate the use of in vivo CLE for endoscopic transsphenoidal surgical procedures.

## Supplementary Materials

The following are available online at https://www.mdpi.com/2077-0383/9/10/3146/s1, Supplementary data 1: Confocal laser endomicroscopy assessment of pituitary tumor microstructure—histopathology questionnaire. Video S1. Confocal laser endomicroscopy video of pituitary adenoma showing sheets of uniform nonlobulated cells with prominent nuclei. Used with permission from Barrow Neurological Institute, Phoenix, Arizona.Video S2. Confocal laser endomicroscopy video loop of pituitary adenoma showing perivascular sheets of the cells. Used with permission from Barrow Neurological Institute, Phoenix, Arizona.
