# Supplementary material for: Confocal Laser Endomicroscopy Assessment of Pituitary Tumor Microstructure: A Feasibility Study
Source: J Clin Med. 2020 Sep 29;9(10):3146. doi: 10.3390/jcm9103146 (PMC7600847; doi:10.3390/jcm9103146)
Supplement: Supplementary file 1 [file jcm-09-03146-s001.pdf]

Supplement 1. Confocal laser endomicroscopy assessment of pituitary tumor microstructure—Histopathology questionnaire

BIOPSY NUMBER ____ All samples are from a mass lesion in the sellar region.

1. Please select statements, that apply to this slide:

_ - The whole folder is non-informative
_ - Mainly normal tissue
_ - Mainly normal tissue, but has some abnormal/suspicious features
_ - Mainly abnormal tissue, unable to characterize
_ - Pituitary adenoma
_ - Other abnormal (list if possible ______)

2. Select histologic features that you identified and used to select the statement in the 1st question

_ - Small organized lobules of pituitary epithelial cells
_ - Uniform nuclei without atypia
_ - Normal tissue architecture, not otherwise specified
_ - Abnormal tissue architecture/growth pattern
_ - Atypical cells
_ - Mitoses
_ - Increased cellularity
_ - Increased vascularity
List any other features: ____________

3. This biopsy is diagnostic of cellular tumor (select one)

1 non-diagnostic
2 less likely diagnostic
3 likely diagnostic
4 more likely diagnostic
5 very diagnostic

4. This biopsy is diagnostic of pituitary adenoma (select one)

1 non-diagnostic
2 less likely diagnostic
3 likely diagnostic
4 more likely diagnostic
5 very diagnostic

5. This biopsy is diagnostic of normal pituitary gland (select one)

1 non-diagnostic
2 less likely diagnostic
3 likely diagnostic
4 more likely diagnostic
5 very diagnostic

6. Rate the quality of the best informative image on a scale from 1 to 5:

1----2----3----4----5
1 poor quality
3 moderate

5 excellent quality

Video 1. Confocal laser endomicroscopy video of pituitary adenoma showing sheets of uniform nonlobulated cells with prominent nuclei. Used with permission from Barrow Neurological Institute, Phoenix, Arizona.

Video 2. Confocal laser endomicroscopy video loop of pituitary adenoma showing perivascular sheets of the cells. Used with permission from Barrow Neurological Institute, Phoenix, Arizona.
